# G protein Gα_q_ subunits engage targets in the nucleus involved in chromatin remodeling and gene expression

**DOI:** 10.1016/j.jbc.2026.111322

**Published:** 2026-02-25

**Authors:** Joseph Loomis, Naincy Chandan, Michael Burroughs, Saji Abraham, Rongxi Zhang, Yiyi Cao, Matthew J. Brody, Gregory G. Tall, Alan V. Smrcka

**Affiliations:** 1 Department of Pharmacology, University of Michigan, Ann Arbor, Michigan, USA; 2 Program in Chemical Biology, University of Michigan, Ann Arbor, Michigan, USA; 3 Nurix Therapeutics, San Franscisco, California, USA

**Keywords:** chromatin, Gq, G Protein, G protein-coupled receptor, nucleus, proximity labeling G protein-coupled receptor

## Abstract

G*α*_q_ is a critical mediator of cell and tissue responses to G_q_-coupled receptor stimulation. Canonically, active G*α*_q_ regulates PLCβ and RhoGEFs. To identify novel G*α*_q_ signaling partners, we performed a proximity labeling proteomic screen in HEK293A cells using TurboID-tagged Gα_q_. Top Gα_q_^(Q209L)^ enriched proteins included known G*α*_q_ interactors (PLCβs, RhoGEFs, and GRK2), supporting the validity of this approach. Also highly enriched were several nuclear proteins including SMARCD3, a component of the SWI/SNF chromatin remodeling complex, and BCAS2, a component of the spliceosome. Luciferase complementation experiments show that Gα_q_ selectively interacts with BCAS2 and SMARCD3 in an activation-dependent manner, and pulldown experiments with purified components demonstrate direct interaction of Gα_q_ and SMARCD3. We also show that a small but significant portion of Gα_q_ is present in the nucleus, and this is increased following GPCR activation or introduction of an activating mutation. Proximity ligation assays indicate that Gα_q_^(Q209L)^ engages SMARCD3 in the nucleus. These data suggest that Gα_q_ engages downstream targets in the nucleus and could therefore directly regulate nuclear processes.

Heterotrimeric G proteins are transducers of a variety of extracellular signals through their association with the intracellular interface of active G protein-coupled receptors (GPCRs). Upon activation, GPCRs act as guanine nucleotide exchange factors (GEFs) for the Gα subunit to promote guanosine diphosphate (GDP) release from Gα-GDP, thereby creating a nucleotide-free Gα subunit (1, 2, 3, 4) that rapidly binds guanosine triphosphate (GTP). GTP binding triggers a set of conformational changes in Gα that lead to dissociation from Gβγ and from the upstream GPCR, thereby leaving Gα and Gβγ primed to engage their respective signaling partners, including second messenger-generating enzymes, GEFs for small GTPases, ion channels, and kinases (2, 5, 6).

Gα_q_-GTP directly interacts with and stimulates PLCβ enzymes, particularly PLCβ1, PLCβ3, and PLCβ4 (7, 8). This results in the hydrolysis of inositol 4,5-bisphosphate into two second messengers, inositol 1,4,5-trisphosphate and diacylglycerol, that ultimately promote intracellular Ca^2+^ mobilization and PKC activation (9). However, Gα_q_-GTP also initiates PLCβ-independent signaling pathways. The best characterized of these is Gα_q_-GTP’s ability to promote RhoA activation by directly activating RhoGEFs, including p63RhoGEF and Trio (10, 11). Other alternative effectors for Gα_q_-GTP have been reported, including, TRPM8, TASK channels, PI3K, p62, and a PKC*ζ*-MEK5 complex (12, 13, 14, 15).

PLCβ and p63RhoGEF’s were identified in targeted searches for transducers that mediate coupling between GPCRs and known downstream signaling pathways. While this targeted approach has successfully identified Gα_q_ effectors, a systematic, unbiased investigation of Gα_q_’s interactome in intact cells has not been conducted. Approaches to screening for protein-protein interactions have typically used yeast two-hybrid, or affinity purification followed by mass spectrometry (16). Such approaches can exclude transient interaction partners due to the use of stringent detergent washes used in affinity based approaches, and they both disrupt cellular context and compartmentation.

Here we used biotin proximity labeling mass spectrometry (PL-MS) to screen for novel Gα_q_ binding partners in intact mammalian cells. By filtering for proteins preferentially enriched in samples from cells expressing mutationally activated Gα_q_^(Q209L)^-TurboID relative to cells expressing Gα_q_^(wt)^-TurboID, we expected to identify targets that preferentially interact with these activation states, as we have shown previously for Gα_i1_, Gα_i2_, and Gα_o_ (17, 18). Results from this screen and subsequent validation experiments reveal activation-dependent Gα_q_ access to the nucleus where it interacts with a distinct pool of effectors responsible for regulating nuclear functions.

## Results

### Design and validation of G*α*_q_-TurboID fusion proteins

To screen for novel, activation-state-dependent Gα_q_ interactors we used a biotinylation proximity labeling based approach adopted previously by our laboratory (17, 19). We generated Gα_q_-TurboID by inserting TurboID—flanked on either side by an SGGGGS linker—between residues F124 and E125 in the alpha helical domain. This internal location was chosen based on similarity to other internally tagged Gα constructs, that retain their functionality (20, 21).

**Figure 1:**
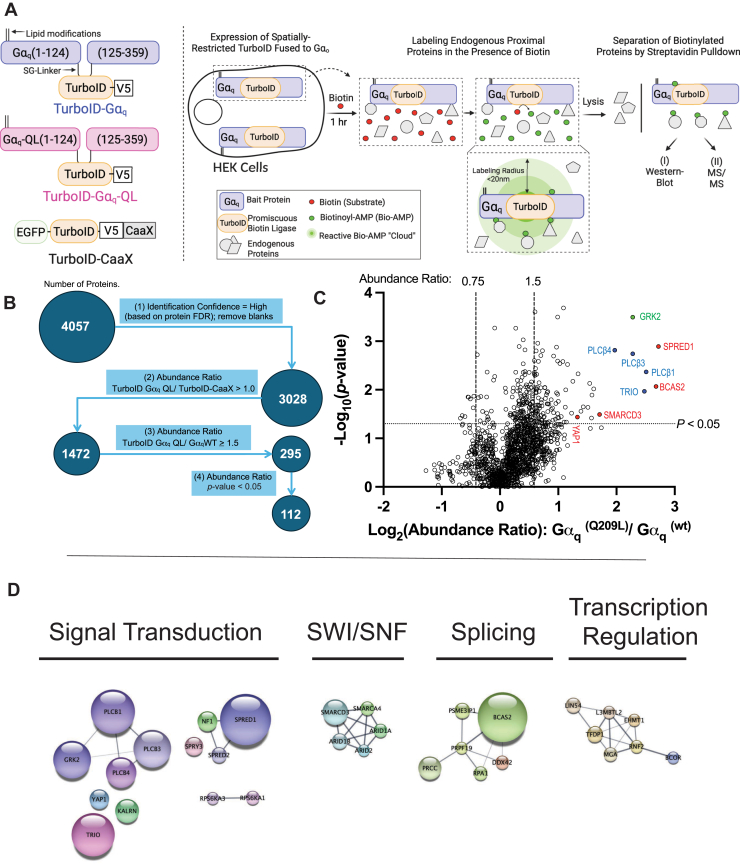
**TurboID-MS screen reveals potentially novel interaction partners for active Gα_q_.***A*, workflow for and design of TurboID-MS experiments. Briefly, HEK293A cells were transfected with either G*α*_q_^(Q209L)^-TurboID, G*α*_q_^(wt)^-TurboID (*right*), or TurboID-CaaX (PM control) in triplicate. After 24 h, cells were incubated with biotin (500 μM) for 60 min. Biotinylated proteins were captured *via* streptavidin pulldown. Samples were subjected to on-beads trypsin digestion and tandem mass tag labeling and LC-MS/MS to identify and quantitate the proteins present in each sample. *B*, flow diagram of TurboID-MS filtering criteria that were used to identify 112 QL-enriched proteins (panel C). *C*, volcano plot depicting TurboID-MS results for proteins that were identified with high confidence and had a normalized Gα_q_-TurboID-QL/TurboID-CaaX abundance ratio greater than 1.0. In the upper right are the 119 classified as G*α*_q_^(Q209L)^-enriched and includes known Gα_q_ effectors (*blue dots*) and a Gα_q_ regulator (*green dot*). Select potentially novel Gα_q_ interaction partners are indicated with *red dots*. *D*, select string clusters based on functional enrichment categories (connected by edges) and manual annotation (not connected by edges). Enriched categories based on GO Terms and functions.

Our experimental design (Fig. 1*A*) exploited differences between Gα_q_^(WT)^ and mutationally active Gα_q_^(Q209L)^ to probe for the active Gα_q_ interactome. The Q209L mutation replaces a catalytic glutamine, blocking the ability of Gα_q_ to catalyze GTP hydrolysis resulting in constitutive activation (22, 23, 24). Accordingly, Gα_q_^(Q209L)^-TurboID stimulated PLCβ activity more strongly than Gα_q_^(wt)^-TurboID in cotransfected COS-7 cells (Fig. S1*A*) demonstrating that the G*α*_q_-TurboID constructs are functional. Incubation of cells expressing Gα_q_^(wt)^-TurboID, Gα_q_^(Q209L)^-TurboID, or a PM localized control (TurboID-CaaX) with 500 μM biotin for 1 h led to biotinylation of multiple proteins (Fig. S1*B*).

### Proximity labeling proteomics identifies both known and novel interactors for active G*α*_q_

Gα_q_-TurboID-MS identified 4057 biotinylated proteins; of these, 3028 were identified with high confidence (as assessed by the protein false discovery rate) and by filtering for proteins with at least two peptide spectral matches, (Fig. 1*B*). We filtered our data to select proteins that were more abundant in G*α*_q_-TurboID-QL samples compared a TurboID-CaaX non-specific control (normalized QL/CaaX abundance ratio greater than 1.0), with normalized abundance ratio (QL/WT) ≥ 1.5 and a *p*-value < 0.05. These 112 proteins were classified as Gα_q_^(Q209L)^ enriched and as potential downstream binding partners. These Gα_q_^(Q209L)^ enriched proteins included known effectors including PLCβ1, PLCβ3, PLCβ4, Kalirin and Trio, (Fig. 1*C*, blue dots in 1C), and a known Gα_q_ binding partner GRK2 (25), (Fig. 1*C*, green dot). Other proteins not known to interact with active Gα_q_ were also Gα_q_^(Q209L)^-enriched, including SPRED1, BCAS2, SMARCD3, and YAP1 (red dots in Fig. 1*C*) (Table S1).

Functional enrichment analysis for the 112 Gα_q_^(Q209L)^-enriched proteins was performed using String Enrichment in Cytoscape, with the total 4057 biotinylated proteins identified as the background reference data set (26). Surprisingly, the top GO cellular component was “nucleoplasm” (FDR 1.2 × 10^−9^) with “transcription regulation” (FDR 7.4 × 10^−8^) and “chromatin regulator” (FDR 7.8 × 10^−7^) also highly ranked (Table S2). 80 of 112 QL-enriched proteins had annotated associations with the nucleus (Fig. S2). Cytoscape software was used to functionally subcluster these proteins, with some added manual clustering based on known functions. Only the major significant clusters are shown in Figure 1*D*. These were then each analyzed individually for functional enrichment (GO or Kegg analysis). Cluster one contained proteins involved in signal transduction (FDR 2.8 × 10^−5^). Cluster two is enriched in components of the SWI/SNF chromatin remodeling complex (FDR 3.6 × 10^−9^). Cluster three proteins are enriched as components of “mRNA Splicing” and nuclear bodies which are distinct nuclear structures involved in active transcriptional processes and “mRNA splicing” (FDR 0.03). Cluster four proteins were enriched in proteins involved in “transcriptional regulation” (FDR 1.4 × 10^−7^).

### Nuclear Gα_q_^(Q209L)^-TurboID enriched proteins preferentially Interact with Gα_q_^(Q209L)^

To confirm that select nuclear-annotated proteins identified by TurboID-MS selectively interact with Gα_q_^(Q209L)^, we adapted a NanoBiT luciferase complementation assay using Gα-LgBiT (27). We chose SMARCD3, BCAS2, and YAP1 as potential Gα_q_ binding partners in part due to their strong enrichment in the Gα_q_^(Q209L)^-TurboID MS samples. SMARCD3 is a component of the SWI/SNF chromatin remodeling complex and BCAS2 is a component of the spliceosome complex. These proteins/complexes are considered as nuclear resident. YAP1 is a transcriptional regulator that can be activated downstream of G_q_ signaling and shuttles between the nucleus and cytoplasm.

We appended the “natural peptide” (NP, K_D_ = 900 nm for LgBiT (28)) onto the C-terminus of SMARCD3, or small bit (SmBiT, K_D_ = 190 μm for LgBiT) onto the C-terminus of BCAS2 and YAP1. As a control NP was appended to the C-terminus of PLCβ4. Since SmBiT and NP have relatively low affinity for LgBiT, complementation is expected to be driven by the affinities for the appended putative target proteins for Gα_q_. These SmBiT/NP fusion proteins were coexpressed with either Gα_q_^(wt)^-LgBiT or Gα_q_^(Q209L)^-LgBiT in HEK293A cells. Confirming the viability of this approach, Gα_q_^(Q209L)^-LgBiT interacted selectively with the known binding partner, PLCβ4-NP (Fig. S3).

**Figure 2:**
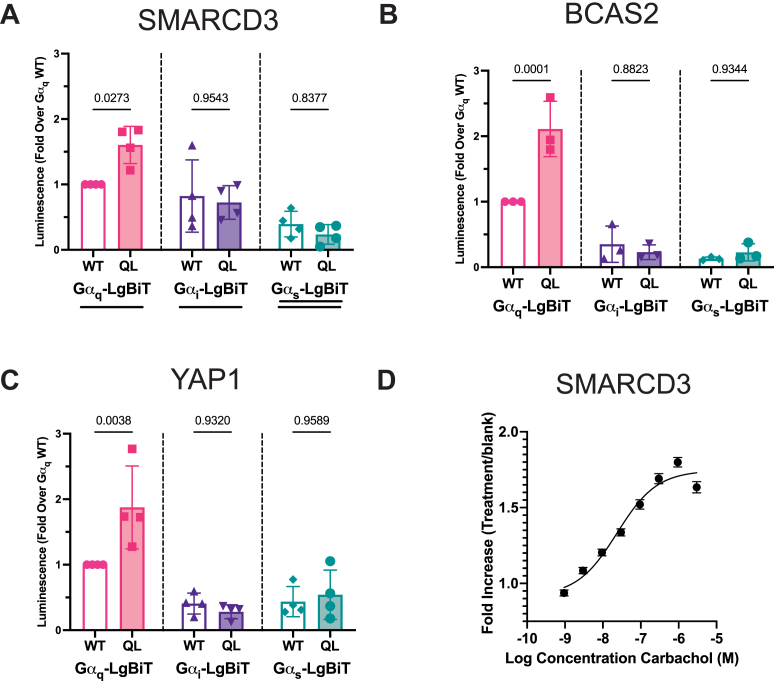
**NanoBiT luciferase complementation assays validate potentially novel Gα_q_ interactors.***A*, NanoBiT complementation assay examining SMARCD3-NP interactions with wt or Q209L forms of Gα_q_, Gα_i1_ or Gα_s_, all fused to LgBiT. Data are expressed as mean fold change (±σ) in luminescence over Gα_q_^(wt)^-LgBiT from N = 3 to 4 independent experiments (n = 4 technical replicates per N). *B*, BCAS2-SmBiT, or (*C*) YAP1-SmBiT NanoBiT complementation were examined as in as in A. *D*, SMARCD3-NP, Gα_q_(wt)-LgBiT and M3 muscarinic receptor were transfected into HEK293 cells, followed 24 h later by stimulation with the indicated concentrations of carbachol for 3h followed by luminescence measurement. N = 3, n = 4. Data were processed as in *A*. Data in *A*, *B* and *C* were analyzed using a one-way ANOVA with Sidak’s multiple comparisons post-test (relevant *p*-values shown). Corresponding Gα_q_-LgBiT and POI-SmBiT-V5 expression blots are for *A*, *B*, and *C* are in Figure S4 (representative of N = 3–4 independent experiments) For all NanoBiT complementation experiments, luminescence from Gα_q_-LgBiT transfected in the absence of a complementing peptide (NP, SmBiT or HiBiT) was less than 1% of the complemented signal.

Concordant with the PL-MS enrichment, SMARCD3-NP preferentially interacted with Gα_q_^(Q209L)^-LgBiT relative to Gα_q_^(wt)^-LgBiT (Fig. 2*A*). No such preference was observed for Gα_i_- or Gα_s_-LgBiT. Similarly, BCAS2-SmBiT, and YAP1-SmBiT also preferentially interacted with Gα_q_^(Q209L)^-LgBiT relative to Gα_q_^(wt)^-, Gα_s_- and Gα_i_-LgBiT (Fig. 2, *B* and *C*). Western blotting confirms that differences in G protein expression levels are not responsible for these results (Fig. S4). These data support the PL-MS results indicating that these proteins, some of which reside in the nucleus, interact preferentially with the active form of Gα_q_.

To determine if GPCR activation increases engagement of Gα_q_ with nuclear targets we chose SMARCD3 as an example nuclear protein. Cells were transfected with the M3-muscarinic acetylcholine receptor, SMARCD3-NP and Gα_q_^(wt)^-LgBiT. Acute stimulation with carbachol (0–30 min, 100 μm) did not increase in Gα_q_^(wt)^-LgBiT interactions with SMARCD3-SmBiT. In contrast, stimulation with carbachol for 3h resulted in a concentration dependent increase in interactions between these proteins (Fig. 2*D*).

### Gα_q_ activation leads to increased accumulation in the nucleus

**Figure 3:**
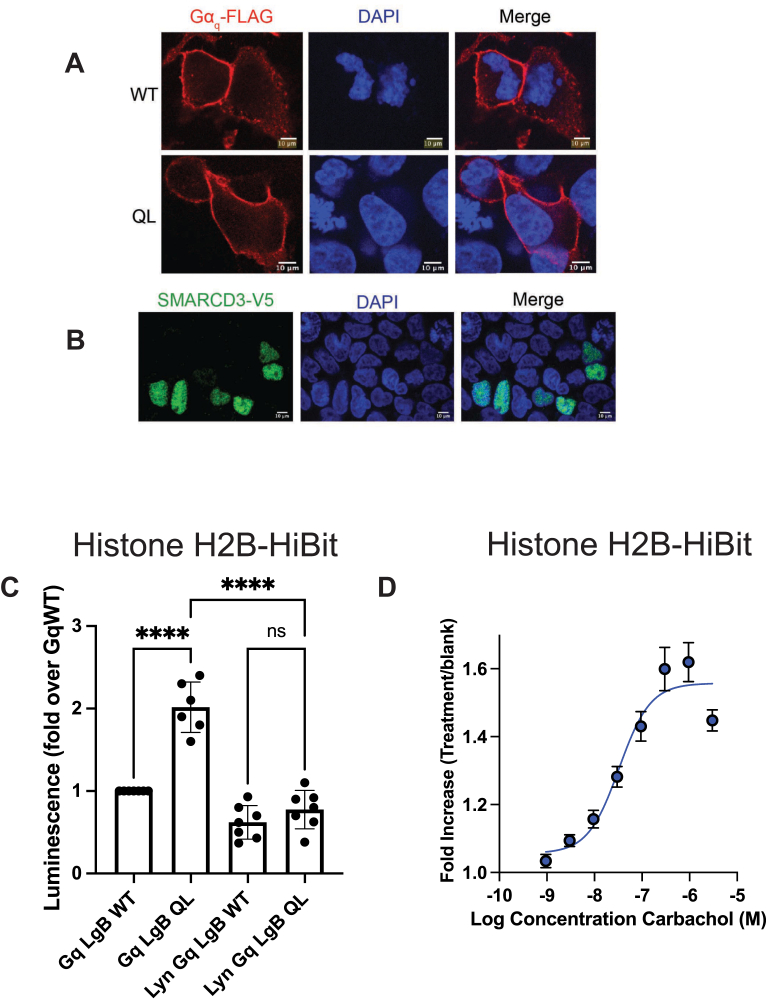
**Activation-dependent Gα_q_ translocation into the nucleus.***A*, f-Gα_q_ localizes strongly to the PM while (*B*) SMARCD3-V5 localizes strongly to the nucleus in HEK293 cells. *C*, constitutive activation of Gα_q_ enhances nuclear residency and is prevented by tethering Gα_q_ to the PM. HEK293 cells stably expressing Histone H2B-HiBiT were transfected with the indicated Gα_q_-LgBiT cDNA containing plasmids. In parallel cells were co-transfected with the indicated Gα_q_-LgBiT cDNAs and GFP-HiBiT to account for differences in expression. Twenty four hour later cells were assessed for luminescence complementation. Data are corrected for differences in expression based on GFP HiBiT. N = 7. Data were analyzed using a one-way ANOVA with Sidak’s multiple comparisons post-test (relevant *p*-values shown). Raw luminescence values: GFP-HiBiT: 20 × 10^6^ ± 5 × 10^6^ (SEM), N = 3 vs. H2B HiBiT: 0.22 ×10^6^ ± 0.08 × 10^6^, N = 7, indicate 1% of the Gα_q_ is present in the nucleus. *D*, activation of M3 muscarinic receptors enhances Gα_q_-LgBiT nuclear accessibility. HEK293 cells stably expressing Histone H2B-HiBiT were transfected with Gα_q_^(wt)^-LgBiT and M3 muscarinic receptor cDNA plasmids. Twenty four hour later cells were stimulated for 3 h with the indicated concentrations of carbachol followed by measurement of luminescence complementation.

We examined the subcellular localization of Gα_q_ and SMARCD3 using immunocytochemistry, and as expected, flag-epitope-tagged Gα_q_ strongly localized to the PM regardless of its activation status (Fig. 3*A*). SMARCD3-V5 strongly colocalized with nuclear 6-diamidino-2-phenylindole (DAPI) staining (Fig. 3*B*). Some weak Gα_q_ staining was also observed in the nucleus (Fig. S5), however, we did not detect a difference in nuclear abundance between Gα_q_^(Q209L)^ and Gα_q_^(WT)^. A limitation is that it is difficult to confidently detect small changes in nuclear abundance quantitatively by immunocytochemistry given the much larger abundance of Gα_q_ in other compartments.

As an alternate approach to detect small changes in nuclear localization quantitatively in an intact cell, we created a stable HEK293 cell line expressing histone 2B-fused to HiBiT (H2B-HiBiT). H2B is a well-established strictly nuclear protein and HiBiT is a peptide with high affinity for LgBiT (K_d_ = 0.7 nm) that is sufficient to promote nanoluc complementation even if the appended proteins do not interact, as long as they are in the same compartment (29). Since histone 2B does not bind to Gα_q_, NanoBiT complementation would indicate localization of Gα_q_ in the nuclear compartment. To normalize the data for differences in expression of Gα_q_-LgBiT, saturating GFP-HiBiT, which is widely expressed throughout the cell, was coexpressed with Gα_q_-LgBiT in parallel transfections to measure the total Gα_q_-LgBiT pool. A significant luminescent signal was seen when Gα_q_^(WT)^-LgBiT was expressed in H2B-HiBiT cells that was approximately 1% of the GFP-HiBiT signal supporting the idea that a small fraction of the total Gα_q_ is in the nucleus in the absence of activation (Fig. 3*C*). Gα_q_^(Q209L)^-LgBiT expression yielded a significantly higher signal, suggesting that active Gα_q_ has greater access to the nucleus than the inactive form. Restriction of Gα_q_ to the PM by fusion of the Lyn PM localization sequence to the amino terminus of Gα_q_ (Lyn-Gα_q_-LgBiT) eliminated the difference between Gα_q_^(Q209L)^-LgBiT and Gα_q_^(wt)^-LgBiT, suggesting that G*α*_q_ release from the PM is required for Gα_q_^(Q209L)^-dependent movement to the nucleus. To examine GPCR-dependent nuclear translocation, M3R was co-expressed with Gα_q_^(WT)^-LgBiT in H2B-HiBiT cells followed by stimulation with carbachol for 3h, revealing a concentration-dependent increase in Gα_q_ in the nucleus measured as complementation with nuclear H2B-HiBiT (Fig. 3*D*).

### Gα_q_^(Q209L)^ selectively engages SMARCD3 in the nucleus in distinct puncta

**Figure 4:**
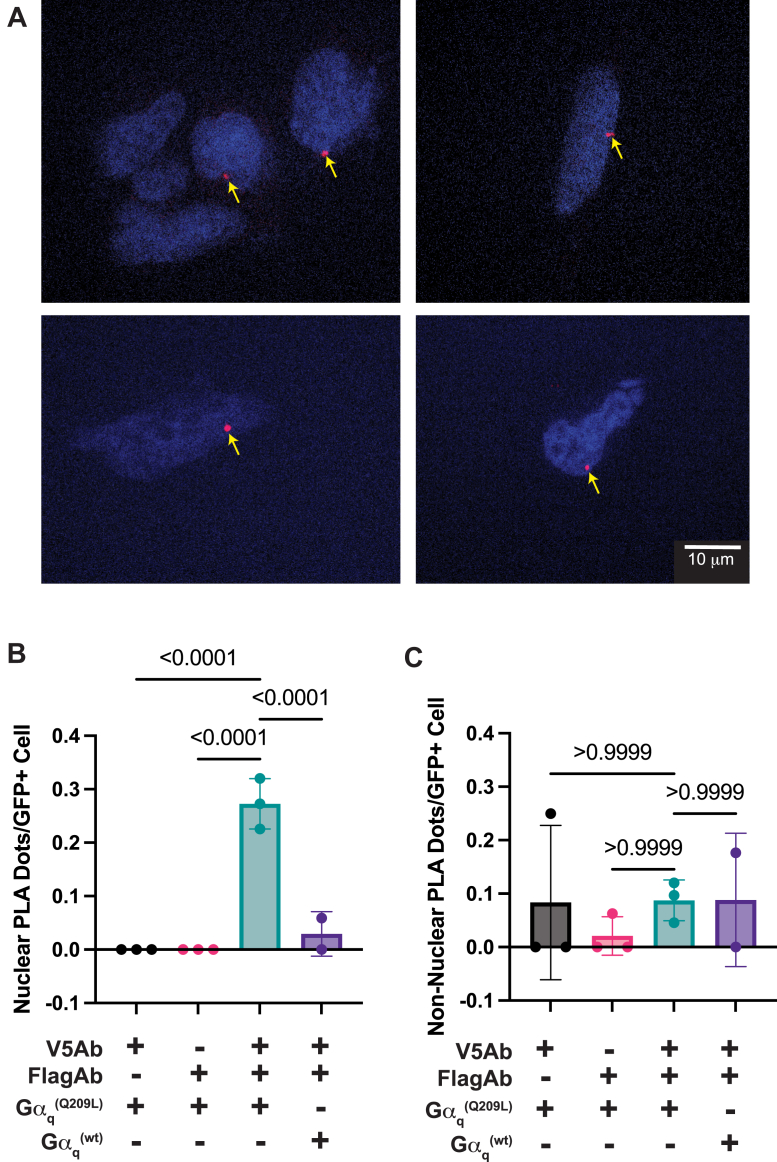
**Gα_q_^(Q209L)^ preferentially interacts with SMARCD3 in the nucleus.***A*, four separate representative images from Proximity Ligation assays performed with HEK293 cells expressing f-Gα_q_^(Q209L)^ and SMARCD3-V5 using Anti-V5 and Anti-Flag antibodies and the Nividia PLA kit. Cells were imaged with Z-stack images collected for each cell. Each image is a confocal slice. Controls with V5 or Flag antibody alone or with Gα_q_^(wt)^ showed very little staining but were quantitated as described in *C*. *B*, the assays for the four conditions, Gα_q_^(Q209L)^ probed with V5-Ab-alone, Gα_q_^(Q209L)^ probed with Flag-Ab alone, Gα_q_^(Q209L)^ probed with both V5-Ab and flag-Ab, or Gα_q_^(wt)^ probed with both V5-Ab and Flag-Ab were performed in N = 3 for Gα_q_^(Q209L)^ (235 total cells sampled) or N = 2 for Gα_q_^(wt)^ (129 total cells sampled). At least 12 separate fields of view were selected for each N based on identification of GFP coexpression in the GFP channel but with relatively low GFP fluorescence. Samples were then blinded and analyzed for the presence or absence of PLA fluorescence in the red channel and the *red dots* colocalizing with DAPI per total GFP expressing cells in the fields were quantified (Table S3). *C*, similar blinded quantitation of the same cells as in *B* except for PLA dots within GFP + cells that did not colocalize with DAPI were counted. Representative control cells where either flag or V5 antibody were omitted are shown in Figure S6.

As was discussed, given the small fraction of Gα_q_ that apparently enters the nucleus and the high expression of Gα_q_ elsewhere in the cell; it is not feasible to use immunocytochemistry to confidently confirm activation-dependent interactions in the nucleus. To circumvent this, we utilized a proximity ligation assay (PLA) to detect and localize interactions between Gα_q_ and SMARCD3, eliminating interference from Gα_q_ that is not in a complex with SMARCD3. Cells coexpressing SMARCD3-V5 and flag-Gα_q_ (f-Gα_q_) (either f-Gα_q_^(Q209L)^ or f-Gα_q_^(wt)^) were fixed and used for this analysis. These epitope-tagged proteins were chosen due the availability of well-defined highly specific antibodies for these epitopes. Interactions were visualized as dots that only occur where co-expressed proteins interact and where both antibodies are incubated with the fixed cells. Cells were also transfected with GFP and the percentage of GFP expressing cells that had dots in the nucleus or at other subcellular locations was quantified. In Figure 4*A* are images of cells expressing V5-SMARCD3 and f-Gα_q_^(Q209L)^. A proportion of the transfected cells had PLA dots (Fig. 4*A*). Qualitatively, cells that did have PLA dots usually had only one or two PLA dots per cell localized at the periphery of the nucleus. Blinded quantitation of these images revealed a significantly higher proportion of the cells expressing Gα_q_^(Q209L)^ had PLA dots compared to cells expressing Gα_q_^(WT)^ (Fig. 4*B* and Table S3). Very few cells had PLA dots at locations other than the nucleus (Fig. 4*C*). Control samples where either the anti-V5 or the anti-Flag antibody were omitted did not result in a significant specific PLA signal (representative cells in Fig. S6) (Fig. 4, *B* and *C*). These data indicate that active Gα_q_ selectively engages SMARCD3 in the nucleus. A speculative explanation for the relatively few, but clear, PLA dots is that active Gα_q_ only engages SMARCD3 in specific nuclear sub compartments.

### SMARCD3 directly interacts with Gα_q_

**Figure 5:**
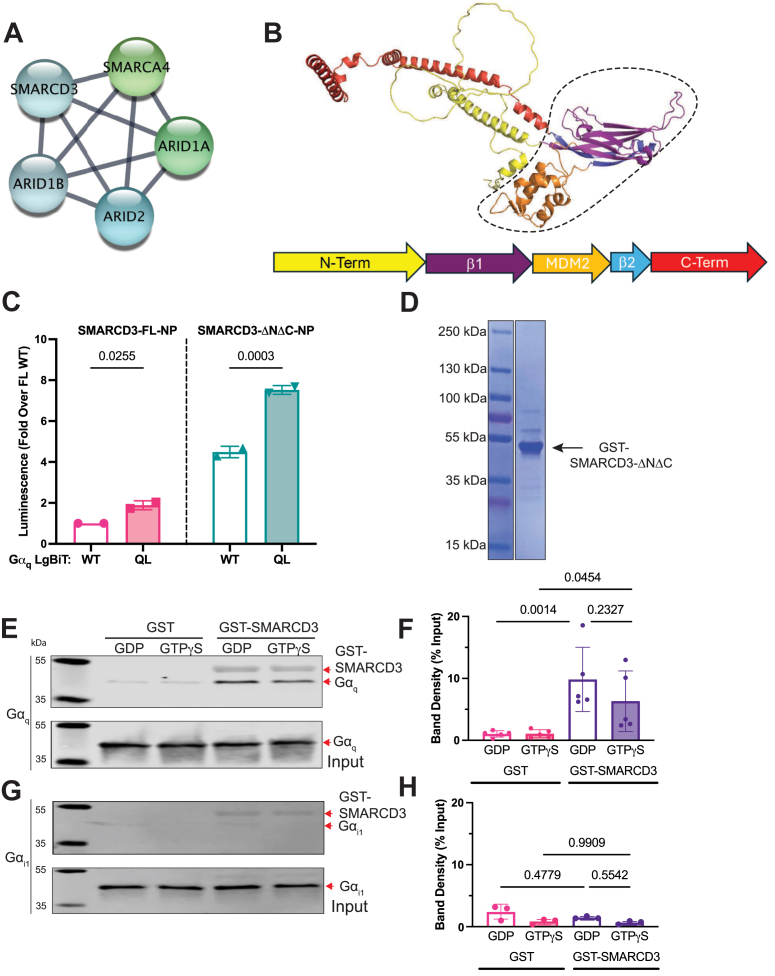
**The “core” of SMARCD3 directly interacts with Gα_q_.***A*, Multiple components of the Swi/SNF complex significantly enriched in Gα_q_^(Q209L)^ TurboID samples (See Table S1). *B*, AlphaFold-predicted model (*top*) and domain map (*bottom*) of SMARCD3 with the “core” (residues M151 to E365) *circled* by the *dashed line*. This core region consists of a β-sheet (β1, *blue*), a SWIB/MDM2 homology domain (*orange*), followed by two beta stands (β2, *violet*) that forms a complete β-sheet through interactions with β1. *C*, NanoBiT complementation assays show that both SMARCD3 NP and SMARCD3(M151-E365) fused to NP (SMARCD3-ΔNΔC-NP) preferentially interact with Gα_q_^(Q209L)^-LgBiT. Data are expressed as mean fold change (±σ) in luminescence over Gα_q_^(wt)^-LgBiT in the presence of SMARCD3-FL-NP from N = 2 independent experiments (*p*-values determined by one-way ANOVA with Sidak’s multiple comparisons test). *D*, SMARCD3-ΔNΔC was inserted into pGex4T2 to generate GST-SMARCD3-ΔNΔC, expressed in BL21(DE3) *E. coli*, and purified *via* glutathione affinity chromatography (Coomassie stain). *E* and *H*, GST pulldowns with purified GST-SMARCD3-ΔNΔC and purified Gα_q_ (*E*) or Gα_i1_ (*G*) show selective SMARCD3 interaction with Gα_q_. Representative immunoblots are shown (note that the ∼50kDa band is a non-specific contribution from GST-SMARCD3-ΔNΔC). *G* and *H*, corresponding Gα band densities in pulldown samples were quantified as a percentage of input Gα. Data are μ ± σ from N = 5 (*E*) or N = 3 (*G*) experiments, respectively; *p*-values were determined using a one-way ANOVA followed by Sidak’s multiple comparisons test. Western blot input controls for all experiments are shown in Figure S7.

SMARCD3 is a component of the SWI/SNF chromatin remodeling complex. In addition to SMARCD3, four other SWI/SNF complex components (ARID1B, SMARCA4, ARID1A, ARID2) (30) were Gα_q_^(Q209L)^-enriched in the TurboID-MS samples, although to a lesser extent than SMARCD3 (Fig. 5*A*, Table S1). To demonstrate direct engagement of Gα_q_ with SMARCD3, we tested binding of purified Gα_q_ to a purified fragment of SMARCD3. Our attempts to purify full length SMARCD3 were unsuccessful. An AlphaFold-predicted model of SMARCD3 (31, 32) (Fig. 5*B*) shows an N-terminus whose structure could not be predicted and is likely to be unstructured (in yellow, poor prediction confidence), a core MDM2 homology region (enclosed in dashed lines, high prediction confidence), and an extended, α-helical C-terminus (in red, poor prediction confidence). We generated a truncation construct lacking both termini, SMARCD3-ΔNΔC, which maintained a statistically significant preferential interaction with Gα_q_^(Q209L)^-LgBiT compared to Gα_q_^(wt)^-LgBiT (Fig. 5*C*); however, this fragment had a large increase in apparent binding to Gα_q_^(wt)^-LgBiT compared to full length SMARCD3.

We purified GST-SMARCD3-ΔNΔC from *E. coli* and conducted binding assays using a glutathione affinity pulldown-based approach (Fig. 5*D*). Both Gα_q_ GDP and Gα_q_ GTP*γ*S complexes bound to GST-SMARCD3-ΔNΔC but not to GST alone (Fig. 5, *E* and *F*). GST-SMARCD3-ΔNΔC did not bind to Gα_i1_ regardless of its nucleotide status (Fig. 5, *G* and *H*). The lack of nucleotide specificity is puzzling and could be the result of using this sub-fragment rather than full length SMARCD3. Nevertheless, there is a clear specific interaction with Gα_q_ indicating that SMARCD3 has determinants for direct engagement with Gα_q_.

## Discussion

Although many Gα_q_ effectors and regulators have been identified since the initial discovery of Gα_q_-PLC*β* in the early 1990s, a systematic, unbiased exploration of the Gα_q_ interactome in intact cells has not been conducted. Here we applied a PL-MS-based approach that we previously applied to Gα_i_ subtypes, to identify potential active Gα_q_ binding partners. Well-established Gα_q_ binding targets were found as top hits, strongly supporting the overall validity of the approach. Surprisingly, the collection of 112 proteins that met the criteria for selective interaction with Gα_q_^(Q209L)^ relative to Gα_q_^(wt)^ is highly enriched in proteins annotated as nuclear-localized proteins involved in processes including chromatin remodeling, transcription and mRNA splicing. Given the relatively small proportion of the total Gα_q_ that appears to enter the nucleus (1–2%), the relative enrichment of nuclear proteins by Gα_q_^(Q209L)^-TurboID is quite striking. We considered the possibility that biotinyl-AMP produced by Turbo-ID has a preference for reactions with nuclear proteins, but this is not consistent with an activation-dependent enrichment, and has not been observed in other PL-MS studies conducted in our laboratory (17, 18, 33). Additionally, if there was a bias for nuclear labeling with turbo ID fused proteins we would expect the total labeled pool of proteins (not filtered for QL vs WT enrichment) would show nuclear enrichment relative to other compartments. String based Cytoscape analysis of functional enrichment of the total Gα_q_ labeled pool (4057 proteins) found “organelle” (3.4 × 10^−262^) (3096 proteins) and cytoplasm (FDR 1.6 × 10^−156^, 2607 proteins) annotated as top “Compartments” compared to “nucleus” well down the list (FDR 3.9 × 10^−88^, 1622 proteins) (Table S4). With 1622 proteins labeled in the nucleus it therefore striking that only a small portion of these were identified as QL enriched. Subsequent validation studies support the PL-MS results and indicate that one destination for Gα_q_ subunits in cells is the nucleus where they engage nuclear proteins.

The Histone 2B HiBiT complementation studies support an activation-dependent mechanism for nuclear accessibility. Gα_s_ signaling at endosomes (34, 35, 36) and the Golgi (37, 38, 39) is well-documented, but how Gα subunits reach these internal compartments to engage GPCRs at these locations is not completely understood. Martin and Lambert showed that Gα_s_ indiscriminately samples various intracellular compartments—most notably the endoplasmic reticulum—upon activation and its endosomal pool is maintained by constitutive endocytosis rather than activity-dependent internalization (40). Similarly, Wright *et al.* recently found that AT_1_R activation leads to the accumulation of G*α*_q_ at early endosomes (and not at the mitochondria), but this accumulation does not involve *β*-arrestin-dependent internalization (41). Partial membrane release upon activation, possibly due to weakened Gβγ interactions and depalmitoylation, has been documented for Gα_q_ (20, 42). Thus, a possible mechanism for this could involve depalmitoylation of Gα_q_, release from the PM, and diffusion to the nucleus. It was surprising to us that we did not detect endosomal or Golgi proteins in our data sets but at the same time these data argue that our results do not simply reflect proximity in the nucleus but rely on specific protein binding interactions. Overall, our findings support the idea that the nucleus is a prominent destination for Gα_q_ where the active state can engage select targets.

Nuclear Gα_q_ localization is not entirely unexpected. There is evidence for G_q_-coupled receptor signaling in nuclei from cardiomyocytes (43, 44, 45) and neurons (46, 47, 48, 49). Similarly, Gβ subunits can localize to the nucleus (50) and recent work by Khan *et al.* demonstrated colocalization and interaction of Gβ_1_ with (nuclear) RNA polymerase II in cardiac fibroblasts treated with angiotensin II (51). Investigations of Gα_q_ signaling mechanisms in these systems have focused on canonical PLC or ERK signaling and have not directly examined Gα_q_ in the nucleus. Our results may bring new mechanistic insights to the signaling consequences of nuclear Gq coupled GPCR activation.

The cellular consequences of Gα_q_-SMARCD3, Gα_q_-BCAS2 or other nuclear Gα_q_ complexes remain to be investigated. In addition to SMARCD3, four other SWI/SNF components (ARID1B, SMARCA4, ARID1A, ARID2) were Gα_q_^(Q209L)^-enriched in our data sets. Gα_q_ interactions with SWI/SNF may have broad implications given that SMARCD3 is involved cardiomyocyte development (52, 53, 54, 55), nutrient signaling (56, 57), vascular smooth muscle cell homeostasis (58), and other physiological functions. One potential system of relevance is uveal melanoma driven by constitutively active Gα_q/11_. In this system PLC*β*-PKC-Ras and Trio-RhoA-FAK pathways, which culminate in MAPK and YAP/TAZ activation, respectively, are required drivers of malignancy (59, 60). It is possible that other non-canonical Gα_q_ interactions, including interactions with the SWI/SNF complexes, are involved in melanoma development, perhaps through altering chromatin accessibility in melanocytes and contributing to malignant transformation. Although further evidence is needed to address this question, recent work suggests that SWI/SNF chromatin remodeling activity is a targetable dependency in uveal melanoma (61). Additionally, inhibiting constitutively active Gα_q_ with FR900359 restores polycomb repressive complex-2 silencing of genes associated with aberrant melanocyte growth and morphology (62). The antagonistic relationship between polycomb repressive complex-2 and SWI/SNF complexes is known to be important in the development of various cancers (63).

Overall, our findings define a pool of proteins whose interactions with Gα_q_ likely occur in the nucleus. Further investigation of these Gα_q_ interactors may reveal novel signaling networks downstream of G_q_-coupled receptors or constitutively active mutants, which will inform efforts to develop targeted therapies for pathologies associated with aberrant G_q_ signaling.

## Experimental procedures

### Plasmid cDNA constructs

#### Proximity labeling

V5-TurboID-Venus-CaaX, Gα_q_-V5-TurboID-WT, Gα_q_-V5-TurboID-Q209L, were synthesized by GenScript. V5-TurboID, along with flanking SGGGGS linkers, was inserted into the alpha-helical domain of Gα_q_ between residues F124 and E125. The final Gα_q_-TurboID construct was organized as follows: Gα_q_(1–124)-Linker-V5-TurboID-Linker-Gα_q_(125–359).

#### Immunocytochemistry

FLAG tagged Gα_q_ constructs (f-Gα_q_^(Q209L)^ and f-Gα_q_^(WT)^) in pcDNA3.1(+) were generated using the Q5 Site-Directed Mutagenesis Kit to insert the FLAG epitope (DYKDDDDK), flanked on either side by SGGGGS linkers, between F124 and E125 of the respective Gα_q_^(Q209L)^ or Gα_q_^(WT)^ cDNAs. The final construct layout was: Gα_q_(1–124)-Linker-FLAG-Linker-Gα_q_(125–359).

#### NanoBiT luciferase complementation

SMARCD3-V5 (HsCD00962738), BCAS2-V5 (HsCD00941964), and YAP1-V5 (HsCD00860804), all in pLenti6.3-V5/DEST, were obtained from the DNASU Plasmid Repository. The natural peptide (NP, GVTGWRLCERILA) or SmBiT (SmB, VTGYRLFEEIL) sequences were inserted after the C-terminal V5-tag in these constructs using the Q5 Site-Directed Mutagenesis Kit (New England Biolabs, E0554S). This resulted in POI-V5-NP or POI-V5-SmB constructs in pLenti6.3-V5/DEST, such as SMARCD3-V5-NP. Plasmid DNAs were amplified using NEB Stable Competent *E. coli* (New England Biolabs, C3040H) grown at 30 °C to avoid DNA recombination difficulties arising from the pLenti6.3-V5/DEST backbone. The Q5 Site-Directed Mutagenesis Kit was used to truncate SMARCD3-V5-NP from the N-terminus to P150, and then from E366 to the C-terminus, resulting in SMARCD3-(151–365)-V5-NP, also referred to as SMARCD3-ΔNΔC-NP. PLCβ4 in pEZYegfp was generated as described previously (64), and the Q5 Site-Directed Mutagenesis Kit was used to insert the NP into the N-terminus, thus resulting in NP-PLCβ4 in pEZYegfp. The following LgBiT constructs—all in in pcDNA3.1(+)—were obtained from Addgene: HA-Gα_q_-LgBiT (#134360), HA-Gα_i1_-LgBiT (#134342), and HA-Gα_s_-LgBiT (#134362). G*α*_q_-LgBiT (lacking the N-terminal HA tag), which was used for all the NanoBiT complementation experiments in this manuscript, was generated by deleting the HA tag in HA-G*α*-LgBiT constructs. With the Q5 Site-Directed Mutagenesis Kit. Constitutively activating QL mutations analogous to Gα_q_-Q209L, Gα_i1_-Q204L, and Gα_s_-Q227L were introduced using the QuikChange II XL Site-Directed Mutagenesis Kit (Agilent, 200523). The N-terminal targeting sequence from Lyn (MGCIKSKRKDGRIPDI) was inserted into the N-terminus of Gα_q_-LgBiT with the Q5 Site-Directed Mutagenesis Kit, resulting in Lyn-Gα_q_-LgBiT. H2B-HiBiT was synthesized by Twist Biosciences and was designed with the HiBiT sequence (VSGWRLFKKIS) followed by the V5 Tag, followed by H2B and inserted into pTwistCMV.

#### GST pulldowns

SMARCD3-ΔNΔC (residues M151-E365) was inserted into the pGex4T2 vector at between the BamHI and EcoRI sites by Twist Biosciences, and the resulting GST-SMARCD3-ΔNΔC construct was synthesized by Twist Biosciences.

#### [^3^H]IP_x_ assays

Gα_q_ in pcDNA3.1(+) was obtained from the cDNA Resource Center. The Q209L substitution was made using the QuikChange II XL Site-Directed Mutagenesis Kit.

### Cell culture

HEK293A cells were obtained from Invitrogen (R70507) and COS-7 cells were obtained from the American Type Culture Collection (ATCC, CRL-1651). Cells were propagated in Dulbecco’s modified Eagle medium (DMEM, Corning, 10–013-CV) supplemented with 10% (v/v) fetal bovine serum (FBS, Thermo Fisher Scientific, FB12999102) and 100 U of penicillin/streptomycin (Gibco, 15140122) at 37 °C with 5% CO_2_. Trypsin-EDTA (Gibco, 25200056) was utilized for passaging cells. Cells with passage number less than or equal to 20 were used for experiments. In experiments involving transient transfection, the propagation media was changed to DMEM supplemented with 10% FBS without penicillin/streptomycin prior to transfection. Unless otherwise noted, Lipofectamine 2000 (Invitrogen, 11668019) was used to transfect cells with the indicated plasmid DNAs at a total μg DNA:μl Lipofectamine ratio of 1:2.5 or 1:3.0.

### Antibodies

The following primary antibodies were used: V5 (Cell Signaling, D3H8Q), HA (Cell Signaling, C29F4), LgBiT (Promega, N7100), FLAG (Invitrogen, PA1-984B), GST (Invitrogen, MA4-004), and Streptavidin-IRDye800 (LI-COR, 925–32230). The following secondary antibodies were also used: Goat anti-rabbit DyLight 800 (Invitrogen, SA535571) and goat anti-mouse IRDye 800CW (LI-COR, 926–32210).

### TurboID proximity labeling for proteomic mass spectrometry

Experiments were performed as detailed previously (17) with minor modifications. Low-passage HEK293A cells were seeded into 175 cm^2^ flasks at a density of 5.5 × 10^6^ cells/flask and incubated overnight. The next day, cells were transfected with 8 μg of TurboID and 4 μg of YFP cDNAs using Lipofectamine 2000 (Invitrogen, 11668019). Approximately 24 h later, transfected cells were treated with DMEM supplemented with 10% FBS and 500 μM biotin for 1 h. Then, the labeling medium was removed, cells were washed twice with 1× PBS and, finally, detached into 1× PBS by mechanical scraping. Scraped cells were pelleted at 4000*g* and 4 °C for 10 min. This scrape-and-pellet sequence was repeated twice more to ensure recovery of the maximum number of cells. Cell pellets were then frozen in liquid N_2_ and stored at −80 °C until further use.

Cell pellets from all conditions were subjected to streptavidin pulldown to isolate biotinylated proteins. These pulldowns utilized fresh stock solutions and low-protein-binding tubes (Eppendorf, 022431081). Pellets were lysed in 1 ml of chilled modRIPA lysis buffer (50 mM tris, 150 mM NaCl, 0.1% SDS, 0.5% sodium deoxycholate, and 1% Triton X-100 [final pH 7.5] supplemented with 1× protease inhibitor [PI] cocktail [P8849, Sigma-Aldrich] and 1 mM phenylmethylsulfonyl fluoride [786-055, G-Biosciences]) for 10 min on ice. 125 units of Benzonase (Sigma-Aldrich, E1014–25KU) were added, and tubes were tumbled at 4 °C for 20 min 0.3% SDS was added to each tube, and samples were incubated for an additional 10 min at 4 °C. Lysates were pelleted at 15,000*g* for 15 min at 4 °C and supernatants were transferred to fresh tubes. The total protein concentration in aliquots from each sample was measured using the Pierce 660-nm protein assay reagent (Thermo Fisher Scientific, 22,660); subsequently, the total protein concentration in each sample was equalized with modRIPA lysis buffer. Five percent of each equalized lysate was reserved for assessing total biotinylation *via* streptavidin Western blot analysis (see below). Meanwhile, 500 μl of a 1:1 slurry of Pierce streptavidin magnetic beads (Thermo Fisher Scientific, 88,817) were added to each of the remaining equalized lysates. Lysates with streptavidin beads were tumbled for 18 h at 4 °C. After this, beads were washed twice with modRIPA lysis buffer and subjected to four stringent washes: 1 m KCl, 0.1 m Na_2_CO_3_, 2% (w/v) SDS in 50 mm Tris pH 7.5, and 2 m urea in 10 mm Tris pH 8.0. Then, beads were washed twice with 1× PBS, frozen with N_2_ (*l*), and stored at −80 °C until further processing (see below).

### Protein digestion, TMT labeling, and LC-MS analysis

These steps were performed by the Proteomics Resource Facility in the Department of Pathology at the University of Michigan.

#### On-beads trypsin digestion

First, samples were reduced with 10 mm DTT in 0.1 m TEAB at 45 °C for 30 min. Second, samples were alkylated with 55 mm 2-chloroacetamide at room temperature (RT) for 30 min in the dark. Third, samples were digested with trypsin (Promega, V5113) at 37 °C. For this, a 1:25 trypsin:protein ratio was used, and samples were subjected to constant mixing in a thermomixer. Proteolysis was stopped with 0.2% TFA, and peptides were desalted with a Sep-Pak C18 cartridge (Waters Corp, WAT036945). Desalted peptides were then dried in a vacufuge and reconstituted in 100 μl of 0.1 M TEAB.

#### TMT Labeling

Reconstituted peptides were tagged with TMT labels using the TMT10plex kit (Thermo Fisher Scientific, 0090110) in accordance with the manufacturer’s protocol. See Table S5 for sample-to-TMT channel information. TMT labeling occurred at RT for 1 h. Labeling reactions were quenched with 8 μl of 5% hydroxylamine (incubated for 15 min), then combined into one sample and dried. This combined sample was split into eight fractions *via* offline fractionation with a high pH reverse-phase peptide fractionation kit (Pierce, 84,868). To prepare for LC-MS/MS injection and analysis, fractions were dried and reconstituted in 12 μl of 0.1% formic acid + 2% acetonitrile.

#### LC-MS/MS analysis

Samples were processed with an RSLC Ultimate 3000 nano-ultra performance liquid chromatography (Dionex) coupled to an Orbitrap Fusion (Thermo Fisher Scientific) mass analyzer. Multinotch-MS3 [REF—McAlister Analytical Chem 2014] was utilized to improve quantitative accuracy. The ultra performance liquid chromatography was equipped with a nano-capillary reverse phase column (PepMap RSLC C18 column, 75 μM inner diameter × 50 cm; Thermo Fisher Scientific). Two microliters of each fraction were resolved on the column at a flowrate of 300 nl/min in the presence of the following 0.1% formic acid/acetonitrile gradient: 2 to 22% acetonitrile in 110 min, 22 to 40% acetonitrile in 25 min, 90% acetonitrile wash for 6 min. The column was re-equilibrated for 25 min in between fraction injections. Resolved fractions were ionized *via* the EasySpray source on the Orbitrap Fusion (Thermo Fisher Scientific). The mass spectrometer collected one MS1 scan (Obitrap; 120K resolution; AGC target 2 × 10^5^; max IT 50 ms) followed by data-dependent, “Top Speed” (3 s) MS2 scans (collision-induced dissociation; ion trap; NCD 35; AGC 5 × 10^3^; max IT 100 ms). The top 10 precursors from each MS2 were fragmented by HCD for multinotch-MS3 prior to Orbitrap analysis (NCE 55; 60K resolution; AGC 5 × 10^4^; max IT 120 ms; 100–500 m/z scan range).

Tandem mass spectra were analyzed using Proteome Discoverer version 2.4 (Thermo Fisher Scientific). For spectral assignment, spectra were searched against the SwissProt human protein database with the following search criteria: MS1 tolerance 10 ppm, MS2 tolerance 0.6 Da, static modifications set to carbamidomethylation of cysteines and TMT labeling of lysine and N-termini of peptides (57.02146 Da and 229.16293 Da, respectively), variable modifications set to oxidation of methionine and deamidation of asparagine and glutamine (15.9949 Da and 0.98401 Da, respectively). Proteins and peptides that passed a 1% false discovery rate threshold were retained for subsequent analysis. TMT reporter ion abundances were quantified using MS3 spectra with an average signal-to-noise ratio of 10 and < 50% isolation interference.

### TurboID-MS sorting criteria

Analyses were performed only on proteins that were identified with high confidence in LC-MS/MS (protein false discovery rate confidence = “high”—these proteins passed a ≤1% FDR threshold using a decoy database strategy for validating MS/MS searches). To account for variation in protein expression between biological replicates, a normalization factor was applied to the raw abundance for each protein in each of our 12 TMT samples. For each TMT sample, this normalization factor was the highest total TMT signal (from TurboID-CaaX N = 3) divided by the total TMT signal in a given condition.

Our key goal in analyzing our normalized data was to identify the Gα_q_ QL interactome—that is, to pinpoint the relatively small subset of proteins that are preferentially enriched in proximity to Gα_q_-TurboID-QL. One challenge in this regard is that TurboID is a powerful proximity labeling approach that can biotinylate any protein with surface-accessible lysine residues, so long as such protein resides within the labeling radius of TurboID. Consequently, the majority of proteins detected in proximity to Gα_q_-TurboID exhibit nearly equal normalized abundances in Gα_q_-TurboID-WT and Gα_q_-TurboID-Q209L samples. Thus, to identify Gα_q_ QL interactome, the following sorting criteria were applied: [1] abundance ratio Gα_q_-TurboID-QL/TurboID-CaaX > 1, [2] abundance ratio Gα_q_-TurboID-QL/Gα_q_-TurboID-WT ≥ 1.5, and [3] abundance ratio *p*-value < 0.05 (calculated using the “ANOVA (Individual Proteins)” feature in Proteome Discoverer).

### Gene ontology overrepresentation analyses

Functional enrichment analyses were performed with the String Protein Interaction Network Function in Cytoscape (65). These analyses identify biological processes that were overrepresented in annotations for various “interested lists” of Gα_q_-TurboID-QL-enriched proteins relative to a “reference list” of all proteins detected with high confidence by TurboID-MS.

### NanoBiT luciferase complementation assays with Gα_q_-, Gα_i_, and Gα_s_-LgBiT

Assays were performed as described previously (18, 33) with minor modifications. Briefly, HEK293A cells were seeded at a density of 3.5 × 10^5^ cells per well in six-well plates (Thermo Fisher Scientific, FB012927) and incubated overnight at 37 °C with 5% CO_2_. The following day, cells were cotransfected with POI-SmBiT (SMARCD3-NP, SMARCD3-ΔNΔC-NP, SMARCD3-SmBiT, BCAS2-SmBiT, YAP1-SmBiT, or NP-PLCβ4) and Gα-LgBiT constructs; then, cells were incubated for approximately 24 h. Transfection media was removed and cells from each condition were washed with 900 μl of warm Hank’s Balanced Salt Solution (HBSS, Gibco, 14,175–095). This wash was reserved in a 15-ml conical tube. Two hundred microliters trypsin-EDTA was added to each well and cells were incubated at 37 °C with 5% CO_2_ for 5 min to facilitate detachment cells. Cells were then resuspended in 900 μl of HBSS, collected in the aforementioned 15-ml tubes, and pelleted (250*g* for 4 min at RT); afterward, the supernatant was carefully removed. Cells were then resuspended in 2 ml HBSS and counted. Subsequently, cells from each condition were pelleted and resuspended in 10 μM furimazine (Aobious, AB36539) in HBSS and 1% DMSO such that there were 500,000 cells/ml. Then, cells were seeded into wells of a 96-well plate (Greiner Bio-One, 655,083) at a density of 50,000 cells/well with n = 4 to 6 technical replicates per condition. The 96-well plate was incubated at 37 °C in a Varioskan LUX plate reader for 5 min, followed by three luminescence measurements in each well. The average of these measurements was used for subsequent data analysis. Following the assay, 400 μl of cells from each condition were collected, pelleted, resuspended in 40 μl of 1X Laemmli sample buffer, and boiled at 95 °C in preparation for protein expression analysis *via* western blotting. For all NanoBiT complementation experiments luminescence from Gα_q_-LgBiT transfected in the absence of a complementing peptide (NP, SmBiT or HiBiT) was less than 1% of the complemented signal.

For H2B-HiBiT experiments, a pool of stable cells was created by transfecting cells in a six well plate with 200ng each of H2B-HiBiT-pTwist-CMV and pcDNA 3.1, followed by selection with 500 μg/ml G418. Surviving cells were pooled and assayed for H2B-HiBiT expression by western blotting. Cells were transfected with the appropriate Gα_q_-LgBiT cDNA plasmids and processed as described above except total Gα_q_-LgBiT expression was controlled for using GFP-HiBiT. Data presented in Figure 2*C* were corrected for differences in expression by dividing the fold changes relative to Gα_q_(wt)-LgBiT in H2B-HiBiT samples by fold differences in expression in GFP-HiBiT samples. No corrections were made in Figure 2*D* since no changes in Gα_q_-LgBiT-GFP-HiBiT complementation were observed in response to carbachol.

### Immunocytochemistry

HEK293A cells were seeded in six-well plates (Thermo Fisher Scientific, FB012927) at a density of 2.5 to 3 × 10^5^ cells/well and incubated over night at 37 °C with 5% CO_2_. Transfection was conducted using Lipofectamine 2000 (Invitrogen) with maximum of 1000 ng total DNA per well. After overnight incubation cells were detached and seeded onto PDL-coated 35 mm glass dishes, after a 3h incubation, 700 μl of culture media was added for overnight incubation. Cells were fixed with 4% paraformaldehyde in PBS (Gibco) for 15 min at RT. Cells were then incubated with 10% normal goat serum in PBS + 0.1% TX-100 buffer (PBS-T) for 1h at RT. Primary antibody was diluted in 2% goat serum in PBS-T and was used to incubate overnight at 4 °C. After washing three × with PBS-T, secondary antibody in 2% normal goat serum in PBS-T at 1:1000 was dispensed to incubate coverslips for 1 to 1.5 h at RT with gentle shaking. Following PBS-T washes, cells were incubated with 4′, DAPI (1000X) in PBST for 15 min at RT, no shaking. Cells were imaged using a Leica DMi8 confocal microscope using a 63× oil lens. Confocal slices were collected and processed with Image J.

### Nuclear extraction

Thermo Scientific NE-PER Nuclear and Cytoplasmic Extraction Kit was used to extract cytoplasmic/membrane vs. nuclear protein fractions from HEK293A cells expressing the indicated cDNAs following the manufacturers protocol. After extraction, the Pierce BCA Protein Assay Kit was used to measure the protein concentration, followed by loading equal amounts of protein, gel electrophoresis and western blotting.

### PLA

*In situ* PLAs were conducted using NaveniFlex Cell Red Kit (NC.MR.100, Navinci) following the manufacturer’s protocol with some modifications. 2.5 × 10^4^ cells were plated on 14 mm coverslips in a 35 mm dish (D11030H, Matsunami). After 24 h, 100 ng of GFP-CaaX, 300 ng Gα_q-_FLAG WT or 150 Gα_q-_FLAG QL with 150 ng PCDNA 3.1, and 200 ng SMARCD3-V5 were expressed for 24 h. The cells were then washed twice with 1 × PBS and fixed with 4% paraformaldehyde in 1 × PBS for 10 min at RT. The cells were blocked for 60 min at 37 °C using the BLOCK reagent provided by manufacturer. ANTI-FLAG M2 mouse (Sigma, F1804) and V5-Rabbit mAb (Cell Signaling, D3H8Q) antibodies were diluted and mixed in Nivacin antibody diluent at 1:200 and incubated overnight at 4 °C in a humidified chamber. For the control condition either the flag or the V5 antibody was left out as indicated. Following the manufacturer’s instructions, the slides were washed three times with TBS-T. Navenibody M1 and Navenibody R2 were diluted 1:40 in Diluent and 80 μl reagent was used to cover the sample area for 60 min 37 °C. After washes, Reaction one was conducted using 1:5 diluted Buffer one and Enzyme one for 30 min at 37 °C. Following with the Reaction two was set up using 1:5 diluted Buffer two and Enzyme two for 90 min at 37 °C in dark. After final washes using TBS 80 μl of Invitrogen SlowFade Gold Antifade Mountant with DAPI media was added to the cells. Cells with green signal on plasma membrane (GFP-CaaX) were picked and Z-stack imaged were acquired for these cells using LEICA DMi8 microscope in confocal mode with a 63 × oil lens and an additional 1.67 × optical zoom. 405 nm excitation was used for imaging of DAPI, 488 nm for GFP-CaaX and 568 nm for PLA dots. Acquisition parameters were kept constant for all the conditions of an experiment. Images were analyzed using ImageJ, samples were blinded and the number of red dots in each GFP + cell were quantified.

### GST-SMARCD3-ΔNΔC purification

GST-SMARCD3-ΔNΔC in pGex4T2 was transformed into BL21(DE3) *E. coli* (Agilent, 200,131) in accordance with the manufacturer’s protocol. A single colony was used to inoculate a 15-ml starter culture in TB media supplemented with 50 μg/ml carbenicillin. This starter culture was grown overnight at 37 °C with shaking at ∼250 rpm. The following day, the starter culture was used to inoculate a 1-L culture in TB media, which was grown at 30 °C until reaching 0.5 < OD_600_ < 0.7. Upon reaching this OD_600_, 100 μM IPTG was added, and the culture was grown overnight at 20 °C and 180 rpm. The next day, BL21(DE3) cells were pelleted *via* centrifugation at 4200*g* for 10 min. The pellet was resuspended in 20 ml of ice-cold lysis buffer (50 mm Tris-HCl pH 8.0, 50 mm NaCl, 5 mm EDTA, 1 mm TCEP, and PIs). PIs included 100 μM PMSF, 21 μg/ml tosyl-L-lysine chloromethyl ketone, 21 μg/ml tosyl-L-phenylalanine chloromethyl ketone, 42 μg/ml tosyl-L-arginine methyl ether, 0.5 μg/ml aprotinin, 0.2 μg/ml leupeptin, 1 μg/ml pepstatin A, and 10 μg/ml soybean trypsin inhibitor. Lysozyme (0.2 mg/ml) was added to the resuspended pellet, followed by incubation on ice for 30 min. Subsequently, cells were further lysed with two, 15-s sonication cycles on ice, and 2 mg DNAse was added, along with 5 mm MgSO_4_. After a 30-min incubation on ice, lysates were pelleted for 20 min at 46,000*g* and 4 °C. The supernatant, which contained soluble GST proteins, was transferred to a clean 50-ml conical tube and passed through a 0.22-micron filter. In the interim, a benchtop gravity column was packed with 1 ml of glutathione sepharose resin (Cytiva, 17075601) and pre-equilibrated with five column volumes of cold lysis buffer. Then, the filtered supernatant was loaded onto the equilibrated column and allowed to flow through to a collection tube. The collected flow-through was passed through the column a second time; then, the column was washed with 10 bed volumes of cold lysis buffer to remove low-affinity interactors. After this, GST-SMARCD3-ΔNΔC was eluted *via* the application of glutathione buffer (10 mm Tris-HCl pH 8.0, 50 mm NaCl, 5 mm EDTA, and 10 mm of freshly made reduced L-glutathione (powder from Sigma-Aldrich, G4251)). Such elution occurred in five 1-ml fractions, which were analyzed for protein content *via* A_280_ measurements and SDS-PAGE with Coomassie staining. Fractions containing GST-SMARCD3-ΔNΔC (molecular weight ∼52 kDa) were pooled and exchanged into storage buffer (50 mm Tris-HCl pH 8.0, 100 mm NaCl, 100 μM TCEP, 1 mm EDTA, and PIs) on a PD-10 column. Aliquots of GST-SMARCD3-ΔNΔC were dispensed into clean 1.5-ml tubes, flash frozen in N_2_ (*l*), and stored at −80 °C until use.

### GST pulldowns

Purified Gα_q_ was diluted to 25 μM in chilled buffer TNNED (50 mm Tris-HCl (pH 7.4), 100 mm NaCl, 0.4% (v/v) Nonidet P-40, 1 mm EDTA, 2 mm DTT) supplemented with 2 mm MgCl_2_ and excess (100 μM) GDP or GTPγS. Samples were incubated overnight at 10 °C to allow for nucleotide binding. Meanwhile, purified G*α*_i1_ was diluted to 1 μM in TNNED supplemented with 2 mm MgCl_2_ and excess (10 μM) GDP or GTPγS and incubated at 30 °C for 1 h to promote nucleotide binding. Separately, GSH-sepharose beads (Cytiva, 17075601) were washed three times with chilled TBS-T (0.1% Tween-20 in tris buffered saline, pH 7.5), resuspended in chilled TNNED to make a 1:1 slurry, dispensed in 20-μl aliquots into clean 1.5-ml tubes, and resuspended in 400 μl TNNED containing 1 μM GST or 1 μm GST-SMARCD3-ΔNΔC. Twenty five microliters aliquots were extracted as representative GST “load” samples for immunoblot analyses, and samples were tumbled for 45 min at 4 °C. Then, GST-protein-loaded GSH-sepharose beads were washed three times with TNNED and resuspended in 200 μl TNNED containing 2 mm MgCl_2_, 10 μm GDP or GTPγS, and purified, GDP- or GTP*γ*S-loaded Gα subunits (see above, [Gα]_final_ = 1 μm). Samples were tumbled at 4 °C for 90 min. After tumbling, 10 μl of each sample was reserved as a representative Gα “input” for immunoblot analyses. Then, beads were washed three times with 500 μl of chilled TNNED. The final wash was removed by dipping a 30-gauge needle into the beads, and beads were resuspended in 48 μl of room-temperature TNNED containing 10 mm reduced L-glutathione (Sigma-Aldrich, G4251) at pH 8.0. These samples were incubated at room temperature for 1 h without tumbling to promote selective elution of Gα-GST-SMARCD3-ΔNΔC protein complexes. Beads were pelleted (1000*g* for 2 min), and each 48-μl supernatant was carefully transferred to a fresh 1.5-ml tube containing 16 μl of 4X Laemmli sample buffer (Bio-Rad, 1610747, with freshly added β-mercaptoethanol) to create an “eluate” sample for immunoblot analyses. All samples—load, input, and eluate from each condition—were heated at 65 °C for 10 min and subjected to immunoblot analyses.

### [^3^H]IP_x_ accumulation assay

Experiments were performed as described previously (64) with some modifications. COS-7 cells were seeded in a 24-well plate (Thermo Fisher Scientific, FB012929) with poly-D-lysine coating at a density of 1 × 10^5^ cells/well. Cells were subjected to reverse transfection with indicated constructs (300 ng DNA total) in accordance with the manufacturer’s protocol for Lipofectamine 2000 (Invitrogen, 11668019). Cells were incubated for approximately 24 h at 37 °C with 5% CO_2_. Transfection media was replaced with Ham’s F-10 Nutrient Mix media (Gibco, 11550043) containing 1.5 mCi/well of myo[2-^3^H(N)] inositol (PerkinElmer, NET114A001MC). After overnight incubation, 10 mm LiCl—an inhibitor of inositol phosphatases—was added to each well and cells were incubated for 1 h at 37 °C with 5% CO_2_. Subsequently, media was aspirated, cells were washed once with chilled PBS, and cells were treated with 300 μl of chilled formic acid for 1 h at 4 °C. Extracted [^3^H]IPs were transferred to a 96-well vacuum manifold in which each well was loaded with AG1-X8 anion exchange resin (Bio-Rad, 1401444). Resin was washed five times with 50 mm formic acid, followed by three additional washes with 100 mm formic acid. [^3^H]IPs was then eluted with buffer containing 100 mm formic acid and 1.2 M ammonium formate. Eluates were transferred to scintillation vials, 4 ml of EcoLume Scintillation Cocktail (MP Biomedicals, 882470) was added to each vial, and samples were mixed *via* inverting capped vials five times. [^3^H]IPs was then quantified by scintillation counting.

### Western blotting

Sample lysates were prepared in 1× Laemmli sample buffer (4× stock from Bio-Rad, 1610747, with β-mercaptoethanol added fresh) and resolved on 4 to 20% Mini-PROTEAN TGX gels (Bio-Rad, 4561096). Developed gels were transferred to a nitrocellulose membrane (GVS North America, 1215458). Membranes were stained with Ponceau S (Sigma-Aldrich, 141194), followed by a wash with TBS-T (0.1% Tween-20 in tris buffered saline, pH 7.5) at RT for 5 min. Membranes were then blocked with 3% (w/v) bovine serum albumin (Thermo Fisher Scientific, BP1600) in TBS-T for 15 to 30 min at RT with constant shaking. Primary antibodies were diluted 1:1000 in TBS-T with 3% (w/v) BSA and 0.1% (w/v) NaN_3_ and applied to blocked membranes for either 90 min at RT or overnight at 4 °C. Then, membranes were washed three times with TBS-T for 5 min at RT, followed by secondary antibody application (1:10,000 in TBS-T with 3% (w/v) BSA) for 60 min at RT. After three more RT TBS-T washes, membranes were imaged on an Odyssey Infrared Imaging System (LI-COR Biosciences).

### Statistical analyses

Analyses were performed in GraphPad Prism version 10.3.1 for Mac (GraphPad, La Jolla, CA; Graphpad.com) using the indicated methods. Specific statistical tests are shown in the figure legends. LI-COR-scanned Western blot images were quantitated using Image Studio Lite (version 5.2; https://www.licorbio.com/image-studio). Figure 1*A* was created with BioRender.

## Data availability

All data are contained within the manuscript and supporting information. The mass spectrometry proteomics data have been deposited to the ProteomeXchange Consortium *via* the PRIDE (66) partner repository with the dataset identifier PXD071839. Any plasmids and recombinant proteins described in this study are available from the corresponding author upon reasonable request subject to a completed Materials Transfer Agreement.

## Supporting information

This article contains supporting information.

## Conflicts of interest

The authors declare that they have no conflicts of interest with the contents of this article.
