# Supplementary material for: G protein Gαq subunits engage targets in the nucleus involved in chromatin remodeling and gene expression
Source: J Biol Chem. 2026 Feb 25;302(4):111322. doi: 10.1016/j.jbc.2026.111322 (PMC13129537; doi:10.1016/j.jbc.2026.111322)
Supplement: Supporting Information [file mmc1.docx]

**Supplemental Figures**


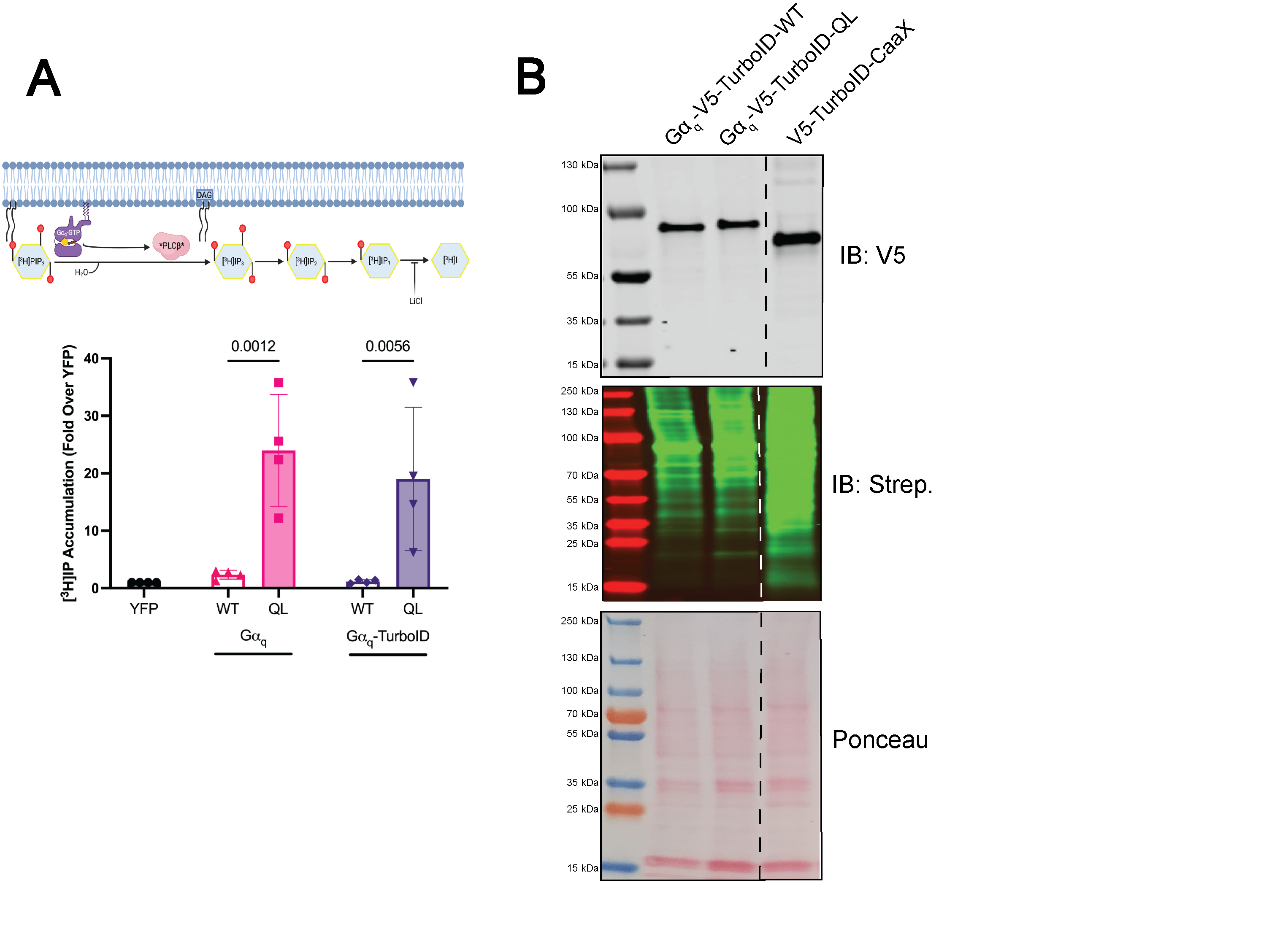


**Figure S1: G𝛼_q_-TurboID constructs are expressed in 293A cells, biotinylate an array of proteins, and are functional**. **(A)** Top: Principle of a well-established tritiated inositol phosphate ([^3^H]IP) accumulation assay for measuring PLC𝛽 activity in mammalian cells (see supplemental methods for details). This assay was used to evaluate G𝛼_q_-TurboID construct function compared to unperturbed G𝛼_q_ controls. Bottom: G𝛼_q_-TurboID-QL strongly activates PLC𝛽 compared to G𝛼_q_-TurboID-WT in transfected COS-7 cells. This parallels the trend observed for unmodified G𝛼_q_ QL and WT. Data are expressed as mean fold change (± 𝜎) over a YFP control and are from N = 4 independent experiments (n = 2 per N). Relevant statistical analyses (see annotated p-values) were performed using a one-way ANOVA with Sidak’s multiple comparisons test. **(B)** Top: Anti-V5 immunoblot showing expression of G𝛼_q_-V5-TurboID-WT, –QL, and V5-TurboID-CaaX, in 293A cells. Middle: Streptavidin blot showing biotinylation of manifold proteins in each condition upon treating transfected 293A cells with 500 µM biotin for 1 hour. Bottom: Ponceau stain provided for loading control. All blots are representative of N = 1 experiment.

**Figure S2:** **Unclustered String Network of Gαq^(Q209L)^ enriched proteins.** Spheres represent nodes and thickness of edges represent the level of evidence for functional or physical interactions. Size of the spheres represents the magnitude of Gα_q_^(Q209L)^ enrichment relative to Gα_q_^(WT)^. Yellow spheres have annotations associated with nuclear localization and/or function. Other colors are for aesthetic purposes.


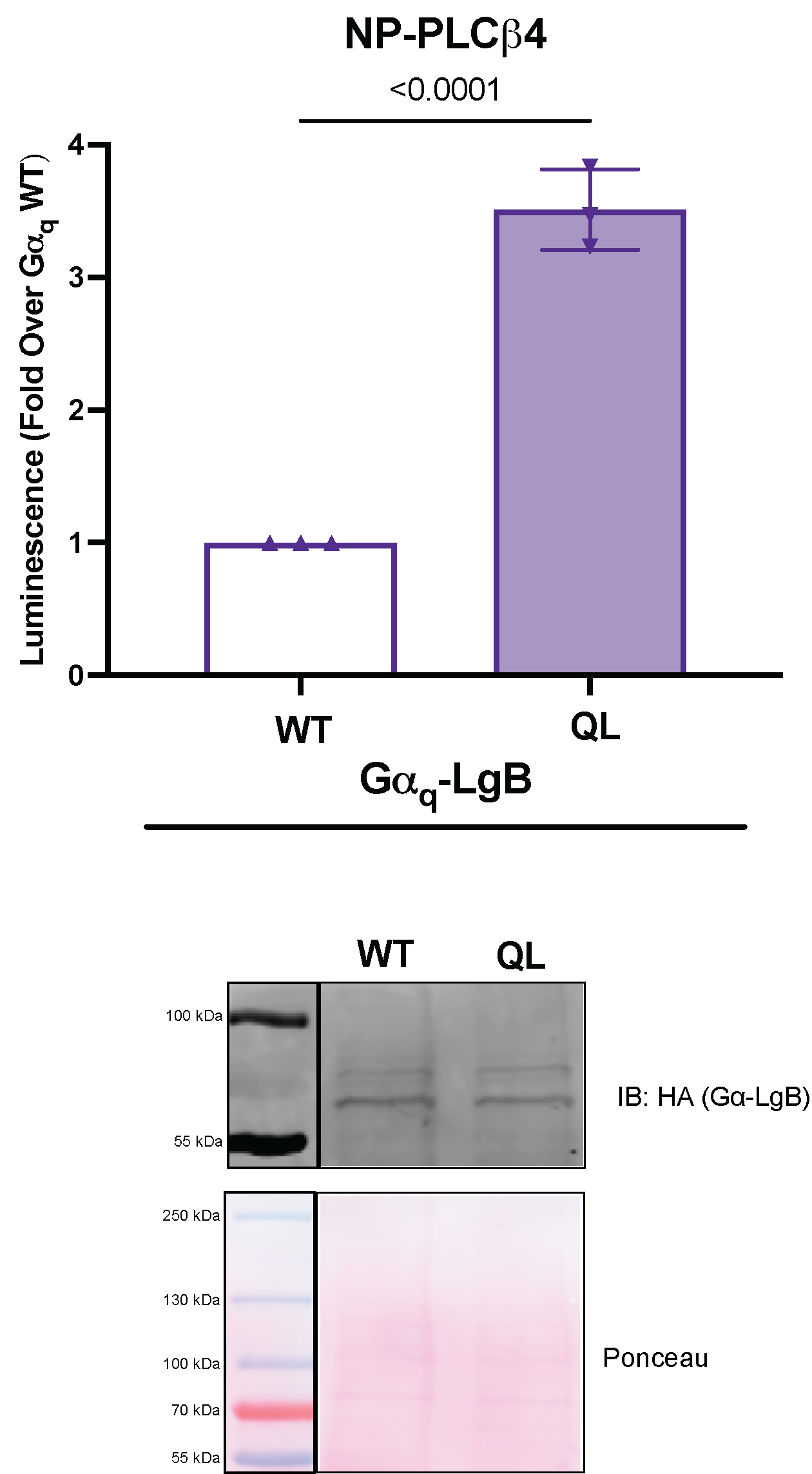


**Figure S3: NanoBiT Luciferase Complementation Assays demonstrate Gα_q_^(Q209L)^ interactions as expected with PLCβ**. PLC𝛽4, a known G𝛼_q_ effector, preferentially interacts with HA-Gα_q_^(Q209L)^-LgBiT in transfected 293A cells. Data from N = 3 independent experiments and are reported as mean fold change (± 𝜎) in luminescence with respect to each respective G𝛼_q_-LgB-WT condition. Statistical analyses were performed with a one-way ANOVA followed by Sidak’s multiple comparison’s test (relevant p-values shown).


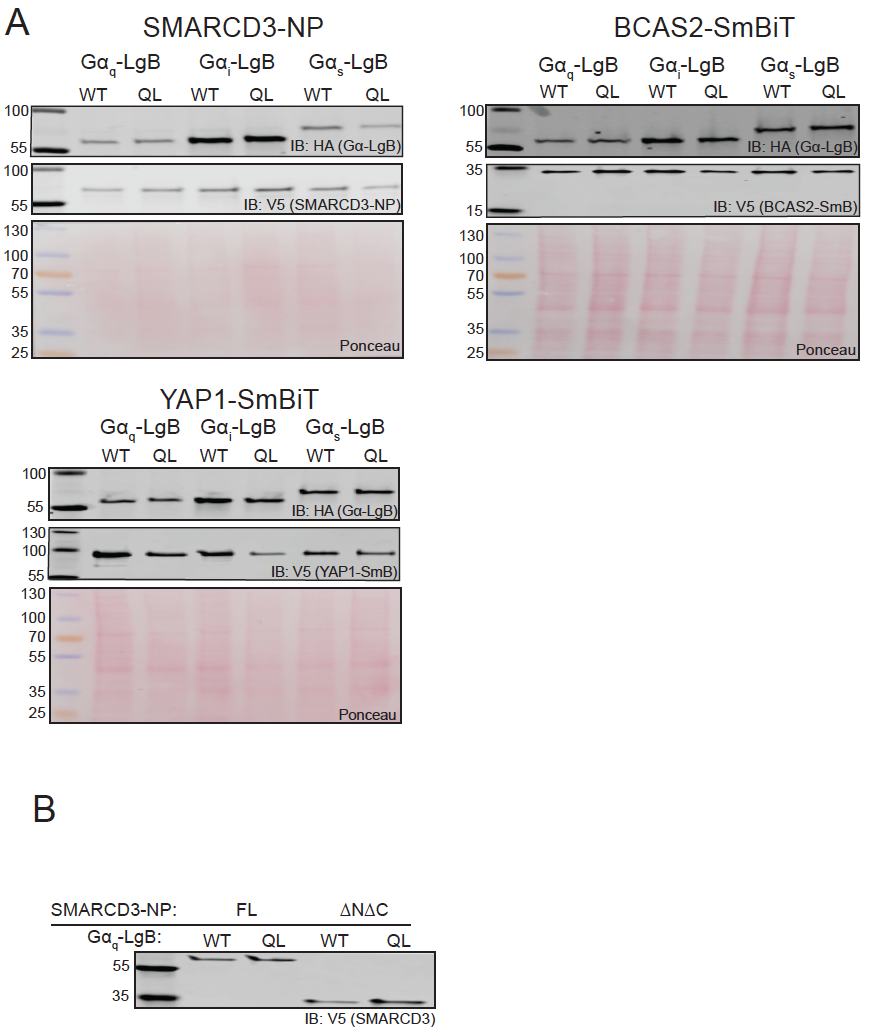


**Figure S4: Protein expression controls.** **(A)** Expression western blots related to Figure 2. **(B)** Expression western blots related to Figure 5C.


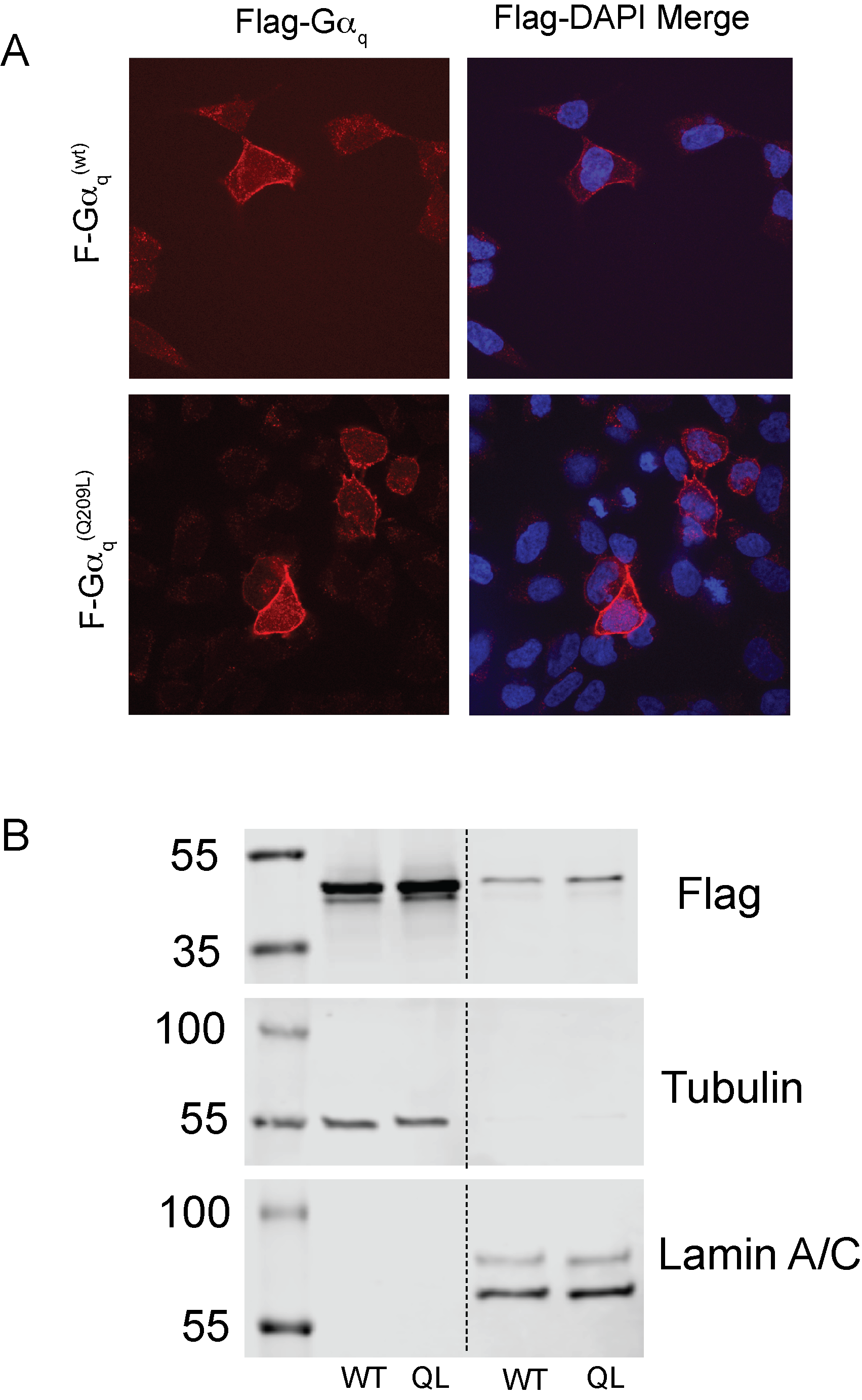


**Figure S5: Immunological analysis of Gα_q_ nuclear localization.** Immunocytochemical staining of HEK293 cells transfected with flag-epitope tagged Gα_q_ (f-Gα_q_). Cells were imaged using confocal microscopy and slice is halfway through the Z stack.

**
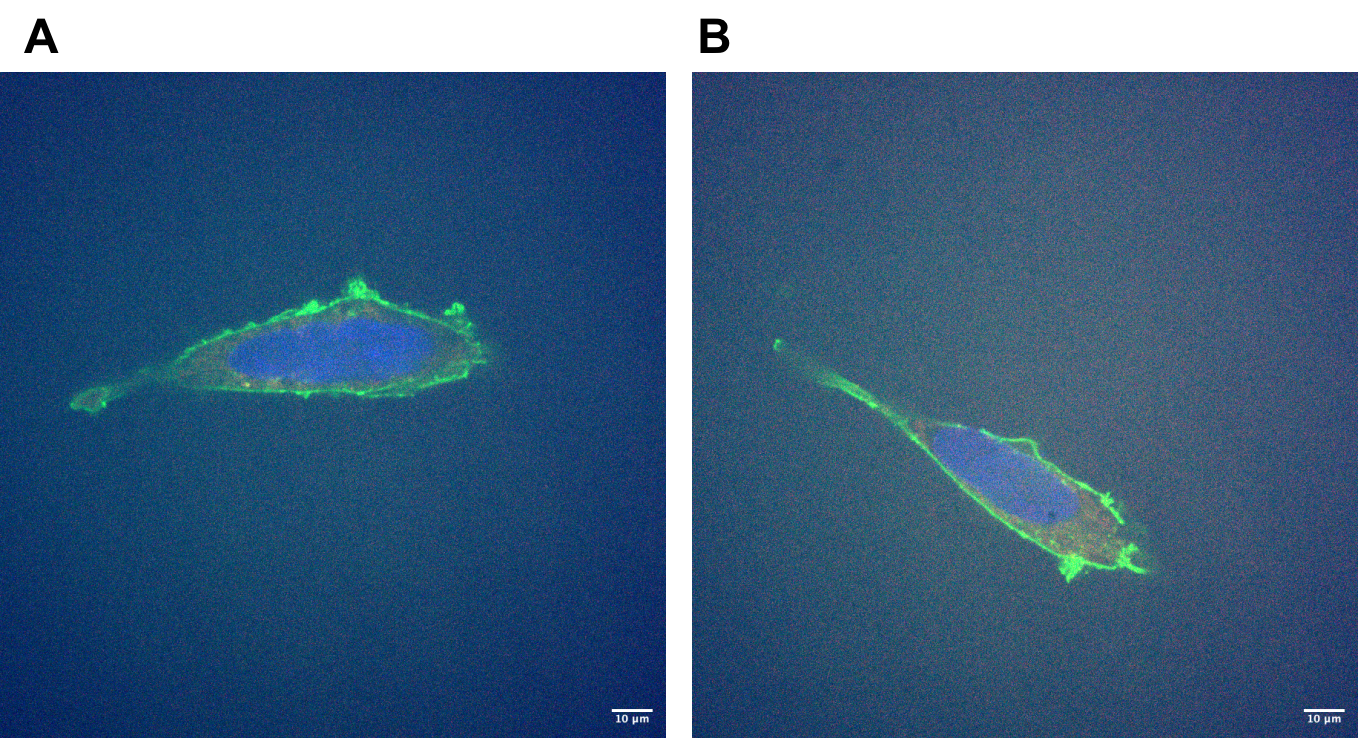
**

**Figure S6:** **PLA Antibody Controls**. Representative images from Proximity Ligation assays performed with HEK293 cells expressing GFP-CaaX to show the plasma membrane, flag-Gα_q_^(Q209L)^ and SMARCD3-V5 using **(A) only Anti-V5 antibody** or **(B) only Anti-Flag antibody** and were treated with Nividia PLA kit. Cells were imaged with Z-stack images collected for each cell. Each image is a confocal image.


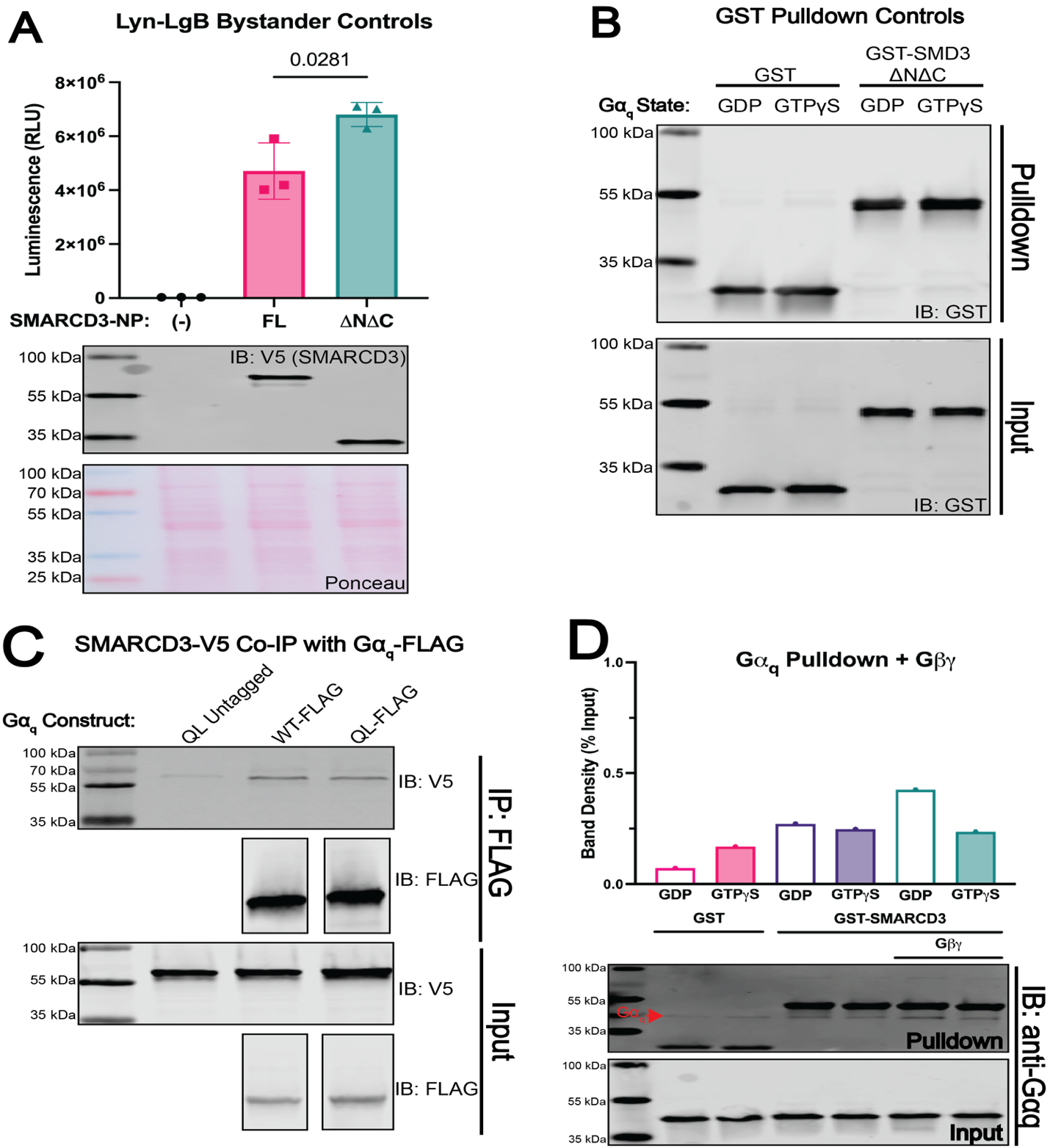


**Figure S7: Protein Expression Controls for SMARCD3-∆N∆C experiments**. **(A)** In transfected 293A cells, SMARCD3-∆N∆C-NP interacts more strongly with Lyn-LgBiT—a plasma membrane bystander NanoBiT control—compared to SMARCD3-FL-NP. Data are µ ± 𝜎 from N = 3 independent experiments (n = 4 per N) and statistical analyses were conducted with a repeated measures one-way ANOVA followed by Sidak’s multiple comparisons test. **(B)** A representative GST immunoblot showing robust pulldown of GST (~26 kDa) or GST-SMARCD3-∆N∆C (~51 kDa) across all experimental conditions.

**Supplemental Tables**

**Table S1: Proteins enriched in Gα_q_^(Q209L)^ TurboID samples relative to Gα_q_^(wt)^ samples** **based on criteria in Fig. 1B.** Proteins are listed based on ranking of relative enrichment in Gα_q_^(Q209L)^/Gα_q_^(wt)^. See Table S1 Excel spreadsheet file.

**Table S2: Cytoscape functional enrichment analysis of Proteins enriched in Gα_q_^(Q209L)^ TurboID samples relative to Gα_q_^(wt)^ samples.** The proteins listed in Table S1 were analyzed for functional enrichment relative to the total pool of 4057 biotinylated proteins identified in the experiment as background.

**Table S3: Blinded data supporting Figure 4B and C.** See Table S2 Excel spreadsheet file.

**Table S4: Cytoscape functional enrichment analysis of all high confidence biotinylated proteins identified.** The total pool of 4057 proteins identified with high confidence were analyzed for functional enrichment relative to the genome as background. Results for “Compartments” are shown.

**Table S5: TMT Labeling Channels for Samples in LC-MS/MS.** See Experimental Procedures for more details.

| Biological Replicate Number | Sample Description | TMT Channel |
| --- | --- | --- |
| 1 | G𝛼_q_-TurboID-WT | 126 |
| 1 | G𝛼_q_-TurboID-QL | 127N |
| 1 | TurboID-CaaX | 129N |
| 2 | G𝛼_q_-TurboID-WT | 130N |
| 2 | G𝛼_q_-TurboID-QL | 131N |
| 2 | TurboID-CaaX | 127C |
| 3 | G𝛼_q_-TurboID-WT | 128C |
| 3 | G𝛼_q_-TurboID-QL | 129C |
| 3 | TurboID-CaaX | 131C |
